# Supplementary material for: Ancient hydrothermal seafloor deposits in Eridania basin on Mars
Source: Nat Commun. 2017 Jul 10;8:15978. doi: 10.1038/ncomms15978 (PMC5508135; doi:10.1038/ncomms15978)
Supplement: Supplementary Information [file ncomms15978-s1.pdf]

File Name: Supplementary Information
Description: Supplementary Figures

File Name: Peer Review File
Description:

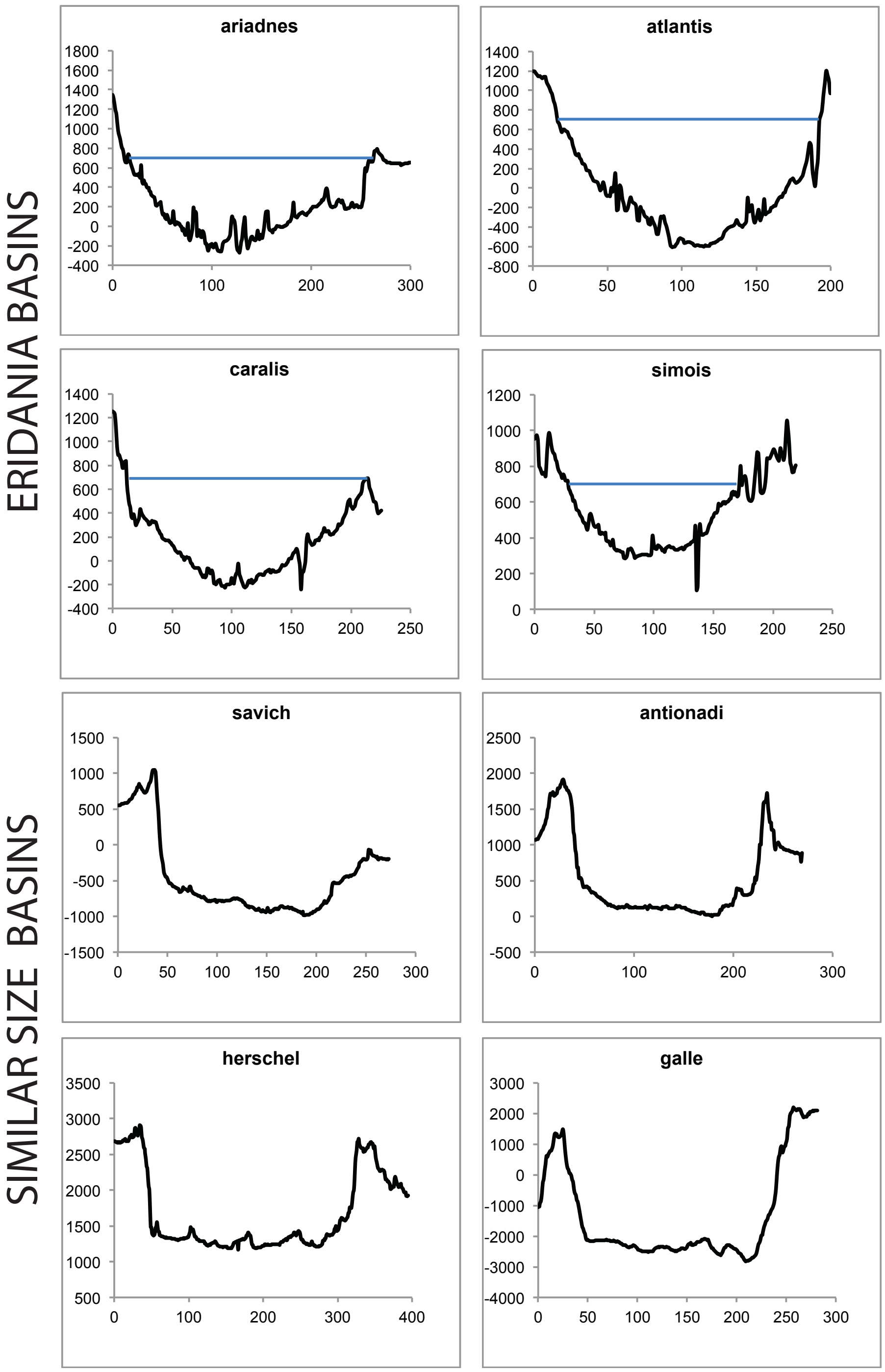

Supplementary Figure 1: The hypsometry of the Eridania sub-basins is unusual for Mars. MOLA elevation data show that the Eridania basins (top four panels) are concave with irregular floor deposits. The 700 m minimum sea level is shown in blue for each basin. The bottom four panels show some similarly sized highland basins. These basins, which have been resurfaced by volcanism and sedimentary infilling in subaerial settings, have completely different topography.

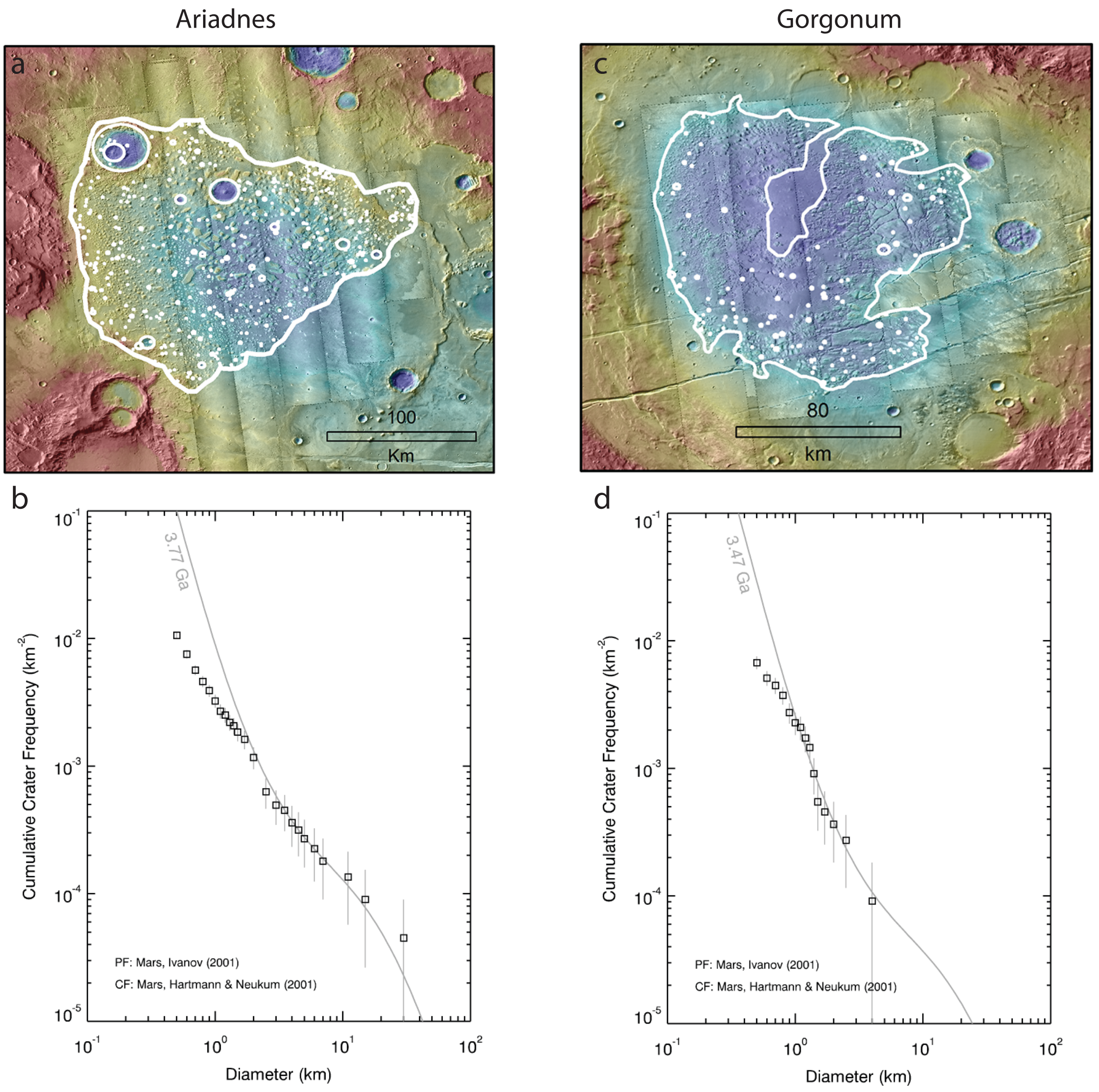

Supplementary Figure 2: MOLA data overlaid on CTX and THEMIS data are shown for Ariadnes colles (a) and Gorgonum chaos (b). In each area, craters 500 m-diametre and larger were counted in over the area of the deep basin, blocky deposits corresponding to chaos and colles. Crater size frequency distributions for are shown for Ariadnes (c) and Gorgonum (d). Curve fits are consistent with minimum surface ages of 3.77 Ga for Ariadnes deep basin deposits and 3.47 Ga for those in Gorgonum.
